# Structural Bases of Coronavirus Attachment to Host Aminopeptidase N and Its Inhibition by Neutralizing Antibodies

**DOI:** 10.1371/journal.ppat.1002859

**Published:** 2012-08-02

**Authors:** Juan Reguera, César Santiago, Gaurav Mudgal, Desiderio Ordoño, Luis Enjuanes, José M. Casasnovas

**Affiliations:** Centro Nacional de Biotecnología, CNB-CSIC, Madrid, Spain; University of North Carolina at Chapel Hill, United States of America

## Abstract

The coronaviruses (CoVs) are enveloped viruses of animals and humans associated mostly with enteric and respiratory diseases, such as the severe acute respiratory syndrome and 10–20% of all common colds. A subset of CoVs uses the cell surface aminopeptidase N (APN), a membrane-bound metalloprotease, as a cell entry receptor. In these viruses, the envelope spike glycoprotein (S) mediates the attachment of the virus particles to APN and subsequent cell entry, which can be blocked by neutralizing antibodies. Here we describe the crystal structures of the receptor-binding domains (RBDs) of two closely related CoV strains, transmissible gastroenteritis virus (TGEV) and porcine respiratory CoV (PRCV), in complex with their receptor, porcine APN (pAPN), or with a neutralizing antibody. The data provide detailed information on the architecture of the dimeric pAPN ectodomain and its interaction with the CoV S. We show that a protruding receptor-binding edge in the S determines virus-binding specificity for recessed glycan-containing surfaces in the membrane-distal region of the pAPN ectodomain. Comparison of the RBDs of TGEV and PRCV to those of other related CoVs, suggests that the conformation of the S receptor-binding region determines cell entry receptor specificity. Moreover, the receptor-binding edge is a major antigenic determinant in the TGEV envelope S that is targeted by neutralizing antibodies. Our results provide a compelling view on CoV cell entry and immune neutralization, and may aid the design of antivirals or CoV vaccines. APN is also considered a target for cancer therapy and its structure, reported here, could facilitate the development of anti-cancer drugs.

## Author Summary

The cell surface aminopeptidase N (APN), a membranebound metalloprotease target for cancer therapy, is a major cell entry receptor for coronaviruses (CoVs), agents that cause important respiratory and enteric diseases. In some CoVs, the virus envelope spike glycoprotein (S) mediates attachment of the virus particles to the host APN protein and cell entry, which is blocked by antibodies that prevent CoV infections. The crystal structures of the S proteins of two porcine CoV in complex with the pig APN (pAPN) or with a neutralizing antibody shown here, reveal how some CoV bind to its cell surface APN receptor and how antibodies prevent receptor binding and infection. The report uncovers a unique virus-receptor recognition mode that engages a glycan N-linked to the pAPN ectodomain, revealing structural determinants of the receptor-binding specificity in CoVs. Neutralizing antibodies target viral residues used for binding to the APN receptor and entry into host cells, showing that efficient CoV neutralization requires immune responses focused toward key receptor binding motifs in the virus envelope. These structural insights, together with the structure of the APN ectodomain, provide a compelling view of relevant cell membrane processes related to infectious diseases and cancer.

## Introduction

The *Coronaviridae* is a large family of enveloped, plus-RNA viruses. They are involved in respiratory, enteric, hepatic and neuronal infectious diseases in animals and humans that lead to important economic losses [1], [2], as well as to high mortality rates in severe acute respiratory syndrome CoV (SARS-CoV) infections [3]. The CoVs are a numerous group of *Coronaviridae*. They have been clustered in the *Coronavirinae* subfamily, which includes three approved genera, *Alpha-*, *Beta-* and *Gammacoronavirus*, as well as a tentative new genus, the *Deltacoronavirus* [4].

Representative CoV species in each genus are *Alphacoronavirus* 1 (comprising transmissible gastroenteritis virus (TGEV), porcine respiratory CoV (PRCV) and related canine and feline CoVs), *Human coronavirus* (HCoV-229E and HCoV-NL63, genus *Alphacoronavirus*), *Murine coronavirus* (including mouse hepatitis virus (MHV), genus *Betacoronavirus*, cluster A), *Severe acute respiratory syndrome-related coronavirus* (SARS-related CoV, genus *Betacoronavirus*, cluster B), *Avian coronavirus* (including infectious bronchitis virus (IBV), genus *Gammacoronavirus*), and Bulbul-CoV (tentative genus *Deltacoronavirus*) [4].

CoV particles display characteristic large surface projections or peplomers (17–20 nm) comprised of homotrimers of the spike glycoprotein (S), a type I membrane protein [1], [5]. The peplomers have a globular portion connected by a protein stalk to the transmembrane domain [6]. The globular region is formed by the N-terminal S1 region, whereas the stalk corresponds to the membrane-proximal S2 region, which mediates virus fusion to host cells and adopts a helical structure characteristic of class I virus fusion proteins [7]. Determinants of CoV tropism locate at the S1 region [1], [8], which mediates attachment of CoV particles to cell surface molecules, initiating virus entry into cells and infection. There is considerable variability in receptor usage among the CoVs. Most *Alphacoronavirus* such as TGEV and HCoV-229E use APN [9], [10], whereas the related HCoV-NL63 uses a distinct cell entry receptor, the human angiotensin converting enzyme 2 (ACE2) [11]; SARS-CoV also recognizes the ACE2 receptor [12]. SARS and NL63 CoV bind to common regions of the ACE2 protein, although the structures of their receptor-binding domains (RBDs) are quite distinct [11], [13]. MHV uses the cell adhesion molecule CEACAM1a [14]; a recent crystal structure showed that the MHV RBD adopts a galectin-like fold [8]. The use of alternative receptors that confer extended tropism has been described for SARS-CoV, MHV and TGEV [1], [8].

The mammalian APNs (CD13) are type II cell surface metalloproteases whose large glycosylated ectodomain has a zinc metal ion at the active site [15]. APN is linked to many cell functions, leading it to be termed the “moonlighting enzyme” [15]. Animal models confirmed a role for this cell surface enzyme in angiogenesis [16]. Peptides and inhibitors that target APN showed a link between this protein and tumor growth and invasion [17], [18]. APN is a target for cancer chemotherapies; drugs that bind this protein have been developed to treat tumors, some of which are in clinical trials [19]. As mentioned above, APN is also a major CoV cell entry receptor [1], [9], [10]. CoV recognition of APN is species-specific, and specificity is associated with N-linked glycosylations in the APN protein [20].

**Figure 1:**
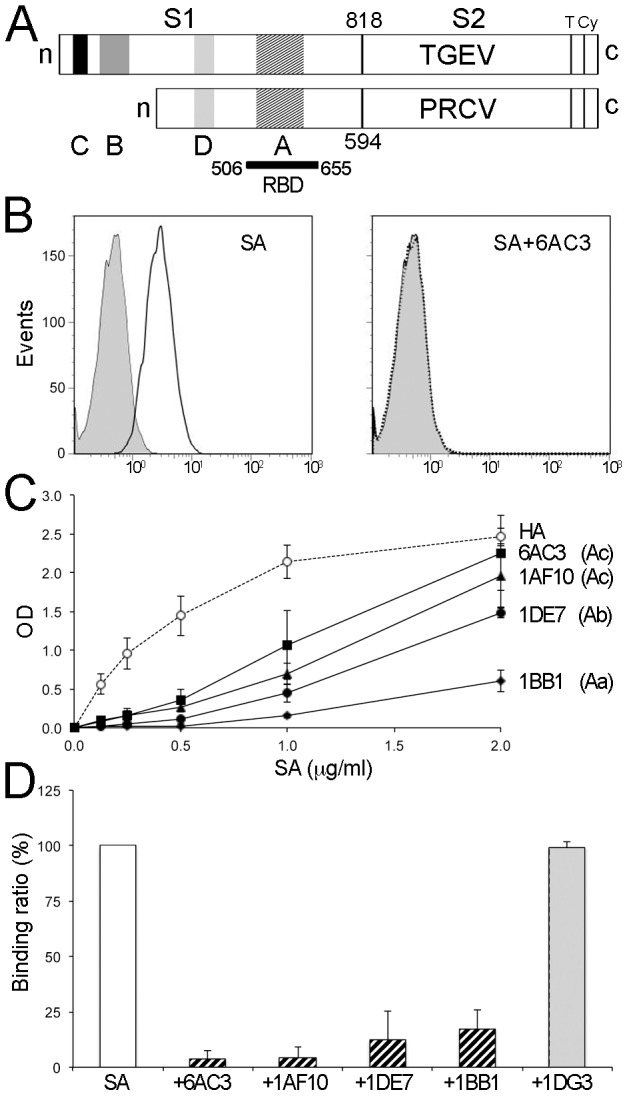
APN-binding domain and epitopes for neutralizing mAbs overlap in TGEV and PRCV S proteins. **A**. Scheme of TGEV and PRCV S proteins showing the S1, S2, transmembrane (T) and cytoplasmic (Cy) regions. Location of the C, B, D and A antigenic sites [21], and the pAPN RBD (bar with N and C-terminal residues) [22] are shown. Length is indicated for mature S1 regions. **B.** A short, soluble S protein variant containing the TGEV RBD region binds to cell surface pAPN. Binding of a bivalent SA-Fc fusion protein (SA) to BHK-pAPN (open histograms), alone (left) or in the presence of the site A-specific 6AC3 mAb (right), as analyzed in FACS. Filled histograms correspond to an unrelated Fc fusion protein. **C.** Binding of site A-specific TGEV-neutralizing mAb to the SA protein. mAb binding to plastic-bound SA protein, monitored by optical density (OD). Site A mAbs are specific for the Aa (1BB1), Ab (1DE7) and Ac (6AC3, 1AF10) subsites [24]. An anti-HA mAb that binds to the HA tag in the SA protein was used as control. **D.** Site A-specific mAbs prevent SA protein binding to pAPN. Binding of the SA protein to BHK-pAPN cells in panel B was monitored alone or in the presence of site A (shown in C) or site D-specific (1DG3) mAb, and the binding ratio determined (see Materials and Methods). **C, D**. Mean and standard deviation for three experiments.

Cell tropism and immune neutralization have been extensively studied in some porcine *Alphacoronavirus*, such as the enteropathogenic TGEV and porcine respiratory CoV (PRCV), a non-enteropathogenic virus derived from TGEV [21]. Both viruses use porcine APN (pAPN) for cell entry. The APN-binding domain in TGEV, PRCV and other *Alphacoronavirus* locates at the C-terminal portion of the S1 region [8], [22], [23], which bears epitopes recognized by CoV-neutralizing antibodies [22], [23], [24], [25]. Most TGEV-neutralizing antibodies cluster at antigenic site A [25], [26], comprised within the RBD at the S1 region (Figure 1A) [22]; the other antigenic sites defined in the TGEV S1 region (B through D) are outside the RBD (Figure 1A) [21].

To date, there is no structural information available on antibody neutralization and APN recognition by *Alphacoronavirus*. We determined crystal structures of the PRCV RBD in complex with the pAPN ectodomain, and the TGEV RBD in complex with the neutralizing monoclonal antibody (mAb) 1AF10 [25]. The RBD adopts a β-barrel fold, with a distinct protruding tip engaged in pAPN recognition. The structures show how these porcine *Alphacoronavirus* recognize its cell entry pAPN receptor and how immune neutralization of these CoVs is achieved by antibody targeting of receptor-binding residues in the S protein. The mechanisms used by TGEV to escape immune neutralization and the evolution of receptor recognition in the CoV family are discussed.

## Results

### APN-binding domain and epitopes for neutralizing mAbs overlap in TGEV and PRCV S proteins

APN receptor recognition and envelope S antigenicity are well documented in TGEV and related PRCV. The pAPN-binding domain was mapped within residues 506 to 655 of the mature TGEV S polypeptide [22], whereas TGEV mAb-resistant (mar) mutants defined four antigenic sites (C, B, D and A) [24], [26] (Figure 1A). Antigenic sites C and B are not present in the PRCV S protein. Antigenic site A determinants are located within the pAPN-binding domain at the C-terminal moiety of the TGEV and PRCV S1 regions (Figure 1A) [21], [22].

We recently reported the modular dissection of the N-terminal S1 region of TGEV and PRCV, and the preparation of soluble S1 length variants with single antigenic sites [27]. We produced a recombinant short S protein fragment termed SA, which comprises only residues 481 to 650 of the TGEV S protein that binds cell surface pAPN (Figure 1B) and displays conformational epitopes for the three antigenic A subsites (Aa, Ab, and Ac) (Figure 1C). Antibodies clustered at the Aa (1BB1), Ab (1DE7) and Ac (1AF10 and 6AC3) subsites blocked binding of the soluble SA protein to pAPN (Figure 1D). The SA protein therefore includes the pAPN-binding domain of TGEV and epitopes for site A-neutralizing mAb. We applied X-ray crystallography to S protein variants containing the RBD of the related TGEV and PRCV, and have identified how these *Alphacoronavirus* bind to the cell surface pAPN and its inhibition by neutralizing antibodies.

### Structure of the TGEV RBD in complex with a TGEV-neutralizing mAb

**Figure 2:**
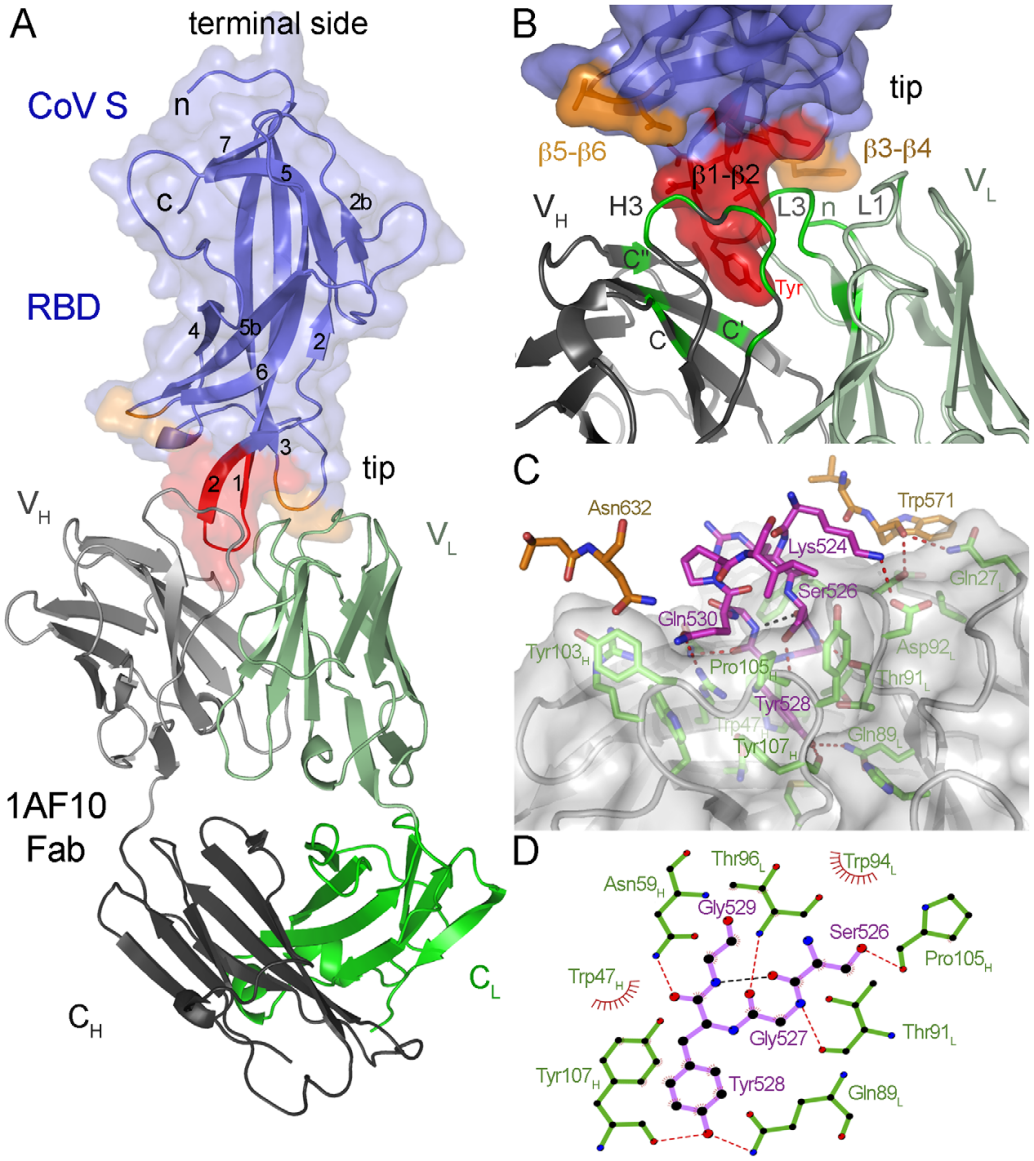
Crystal structure of the TGEV RBD in complex with the TGEV-neutralizing mAb 1AF10. **A.** Ribbon diagram of the Fab fragment of the 1AF10 mAb bound to the TGEV RBD. Blue surface and ribbon representation is shown for the RBD domain, with β-strands and the terminal ends (n and c) where the remaining CoV S locates labeled. The antibody-binding regions at the tip are colored in red (β1–β2 region) and orange (β3–β4 and β5–β6). The 1AF10-Fab fragment is shown with heavy chain in grey and light chain in green. Variable (V_H_ and V_L_) and constant (C_H_ and C_L_) Ig domains of the Fab fragment are also indicated. **B.** Detailed view of the interaction, showing the RBD tip and the RBD-binding regions of the Fab variable domains. Buried regions are colored as in A for the RBD, and in dark green for the mAb. **C.** The RBD epitope for 1AF10. Surface and ribbon representation of the 1AF10 variable domains and stick drawing of buried residues at the interface, shown with carbons in orange or magenta for the RBD, and in green for the 1AF10 mAb. In this and following figures, nitrogens are in blue and oxygens in red, intermolecular hydrogen bonds are shown as dashed red lines, whereas intramolecular hydrogen bonds are black. **D.** Ligplot representation of 1AF10 mAb interaction with the RBD β1–β2 turn. RBD residues in the turn (magenta) and interacting mAb residues (green).

We attempted crystallization of the soluble pAPN-binding SA protein derived from the TGEV S, alone and in complex with several neutralizing mAbs. Crystals were prepared with the SA protein in complex with the Fab fragment of the 1AF10 mAb [25]; the structure of the complex was determined and refined using diffraction data extending to 3.0 Å resolution (Materials and Methods; Table 1). The asymmetric unit of the crystals contains two antibody-RBD complexes, one of which is shown in Figure 2. Residues Pro507 to Val650 of the TGEV S protein, previously identified as the pAPN-binding domain (Figure 1A) [22], were well defined in the crystal structure. They folded in a single domain structure, the RBD of TGEV (Figure 2A). The RBD adopts a β-barrel fold formed by two β-sheets with five β-strands each (scheme in Figure S1A). N- and C-terminal ends are on the same side of the domain (terminal side), which presumably lies close to other S protein domains; at the opposite side, two β-turns (β1–β2 and β3–β4) form the tip of the barrel (Figure 2A), where the mAb binds to the RBD.

**Table 1:** Data collection and refinement statistics.

	TGEV RBD-1AF10	PRCV RBD-pAPN
**Data Processing**		
Space group	P2_1_	C2
Cell dimensions		
*a*, *b*, *c* (Å)	82.77, 106.05, 103.52	220.86, 87.94, 176.91
α, β, γ (°)	90, 95.13, 90	90, 90.54, 90
Wavelength	0.97934	0.97914
Resolution (Å)	25-3.0	25-3.2
*R*_sym_ or *R*_merge_	7.4 (35.4)	5.7 (37.7)
*I*/σ *I*	8.4 (2.0)	5.5 (2.1)
Completeness (%)	95 (95)	96 (97)
Redundancy	5.6 (5.2)	3.0 (3.1)
**Refinement**		
Resolution (Å)	25-3.0	25-3.2
No. reflections	30645	53840
*R*_work_/*R*_free_	21.6/25.1	20.1/24.5
No. atoms		
Protein	8862	16697
Carbohydrates	126	365
Ligands	8	2
Water	2	3
*B*-factors		
Protein	82	95
Carbohydrates	122	125
Ligands	64	75
Water	61	60
R.m.s deviations		
Bond lengths (Å)	0.005	0.003
Bond angles (°)	1.207	0.880

The crystal structures contain two independent molecules in the asymmetric unit. Ligands included in the structures are two acetate molecules in the TGEV RBD-1AF10 structure and zinc ions in the pAPN structure, one in each molecule of the asymmetric unit. Statistics for the highest-resolution shell are in parentheses.

The immunoglobulin (Ig) variable domains of the mAb heavy (V_H_) and light (V_L_) chains contact the β1–β2, β3–β4 and β5–β6 regions of the TGEV RBD (Figure 2B), burying a virus protein surface of ∼810 Å^2^. The buried surface of the 1AF10 mAb is ∼750 Å^2^, with equal contribution by the V_H_ (51%) and V_L_ (49%) Ig domains. Complementarity determining regions (CDR) of the antibody heavy (H3) and light (L1 and L3) chains, the N-terminus of the light chain and the C, C′ and C″ β-strands of the V_H_ domain contact the viral RBD tip (Figure 2B). The CDR-H3 of the 1AF10 mAb is relatively long, with two-residue insertion (Tyr103_H_ and Asp104 _H_) relative to other homologous H3 loops in reported mAb structures (Table S1).

The RBD β1–β2 hairpin with Tyr528 at its tip is at the center of the interacting surface and penetrates between the V_L_ and V_H_ Ig domains of the 1AF10 mAb (Figure 2B and 2C). Similar antibody-antigen recognition is described for some peptides and is common for small hapten molecules [28], [29]. The RBD β1–β2 region contributed 73% of the RBD surface buried by the 1AF10 mAb, and docked between the 1AF10 mAb variable domains (Figure 2B). The β-turn is fully buried between the mAb Ig domains (Figure 2C), forming a contact network with mAb residues (Figure 2D). The RBD residue Tyr528 at the bottom of the pocket contacts mAb residues Trp47_H_ and Tyr107_H_, whereas its hydroxyl group is hydrogen bonded to the side chain of Gln89_L_ and main chain carbonyl of Tyr107_H_ (Figure 2C and 2D). These structural findings on 1AF10 recognition of the RBD β1–β2 region correlate with 1AF10 mAb binding to peptides (MKRSGYGQPIA533) that include this hairpin region [24].

The RBD β3–β4 and β5–β6 regions are at the periphery of the epitope (Figure 2B); their contribution to interaction with 1AF10 is smaller than that of the β1–β2 region, representing respectively ∼17% and 10% of the RBD surface buried by the mAb. They contact either the V_L_ or V_H_ Ig domains (Figure 2B). RBD residues Leu570 and Trp571 at the β3–β4 loop contact the N-terminus, CDR-L1 and CDR-L3 of the V_L_ domain, whereas the β5–β6 loop contacts the long CDR-H3 loop (Figure 2B and 2C).

### Structure of the PRCV RBD bound to the pAPN ectodomain

**Figure 3:**
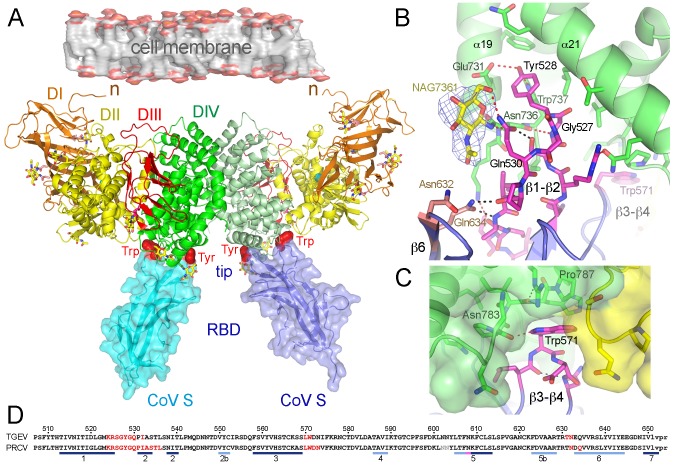
Crystal structure of the PRCV RBD bound to the pAPN ectodomain. **A.** The dimeric PRCV RBD-pAPN complex in the crystals. Ribbon drawings of the pAPN molecules are shown with domains (D) in orange (N-terminal DI), yellow (DII), red (DIII) and green (C-terminal DIV), and the N-terminal end (n) near the cell membrane. The RBD is shown as ribbon and surface drawings in blue and cyan, with the pAPN-binding Tyr and Trp residues at the RBD tip in red. Location of the remaining CoV S is indicated at the RBD terminal end. Glycans are shown as sticks with carbons in yellow and the zinc ion at the pAPN catalytic site as a cyan sphere. **B.** Detail of the RBD β1–β2 region with the exposed Tyr residue interacting with the pAPN. Side chains of RBD and pAPN residues engaged in the interaction are shown as sticks with carbons in magenta or green, respectively. NAG7361 glycan N-linked to pAPN Asn736 is shown with carbons in yellow and the electron density map, determined without the glycan, shown as a blue mesh contoured at 3 sigma. **C.** Detail of the RBD β3–β4 region with the Trp residue interacting with the pAPN. In **B** and **C**, RBD residues are numbered following the TGEV sequence shown in **D**, and intermolecular hydrogen bonds are shown as dashed red lines. **D**. Structure-based sequence alignment of the TGEV and PRCV RBDs. β-strands are marked with bars. TGEV sequence is numbered. In red, 1AF10 mAb- (for TGEV) and pAPN receptor-binding residues (for PRCV) identified by the structures. Residues absent in the RBD structures are in grey, and the thrombin recognition sequences at the end of recombinant porcine CoV RBDs are in lowercase letters.

To characterize CoV attachment to its APN receptor, we attempted crystallization of the pAPN ectodomain in complex with TGEV and PRCV S protein variants comprising their RBDs (Materials and Methods). Crystals were obtained only with a mixture of a PRCV S protein (S3H) and the pAPN. Using these crystals, we determined the structure of the PRCV RBD-pAPN complex by molecular replacement using previously solved structures of the TGEV RBD shown in Figure 2 (97% sequence identity) and of the pAPN ectodomain (Materials and Methods and Table 1). The asymmetric unit of the crystals contained two macromolecular RBD-pAPN complexes (Figure 3A). The PRCV RBD adopts a β-barrel fold like the TGEV RBD (Figure S1). Each pAPN molecule was engaged by the tip of a single PRCV RBD molecule, which bears two exposed aromatic residues (Tyr and Trp) (Figure 3A, in red), and they bound to a membrane-distal region of the pAPN ectodomain (Figure 3A). The RBD N- and C-terminal ends and the remaining CoV S are also distant from the pAPN, and are unlikely to contact the receptor molecule. Based on a cryo-EM structure of the SARS-CoV S [6], the RBD must be also at the viral-membrane distal side of the S and therefore, the receptor binding edge must be accessible for CoV binding to the APN receptor.

The pAPN is a type II membrane protein and the N-terminal end of the ectodomain must be near the cell membrane (Figure 3A). The 25 N-terminal residues of the crystallized pAPN ectodomain are largely disordered in the structure and they might form a flexible region close to the cell membrane. The pAPN ectodomain is composed of four domains (Figure 3A). Domain I (orange) is made of β-strands, domain II (yellow) adopts a thermolysin-like fold bearing a zinc ion at the catalytic site, domain III (red) is a small β-barrel domain, and the C-terminal domain IV (green) is composed of alpha-helices (domain boundaries are shown in Figure S2). The pAPN molecule structure is closely related to that of the human endoplasmic reticulum aminopeptidase-1 [30], [31] (root-mean-square deviation of 2.3 Å for 791 residues sharing 33% sequence identity, based on DALI server). Domain II bearing the enzyme active site is the most related domain (47% identity), whereas domain IV is the most distinct (22% identity). The zinc ion is coordinated to conserved residues at the pAPN active site in domain II (Figure S2). The active site conformation is similar to that of other aminopeptidases (Figure S3). The pAPN crystallized in complex with the PRCV RBD had an open conformation [30], [31], [32], in which domain IV was ∼20–25 Å from domains I and II; this creates a central cavity in which the zinc ion at the catalytic site is highly accessible (Figure 3A).

The mammalian APNs are cell surface metalloproteases that form membrane-bound dimers [33]. The crystallized pAPN ectodomain also behaved as a dimer in solution (Figure S4). The pAPN dimeric assembly showed in Figure 3A buried a large accessible surface (∼980 Å^2^) in each monomer. The dimerization surface comprises 29 residues spread across domain IV, which are distinct from those recognized by CoV (Figure S2). Similar dimeric assemblies were observed in two crystal structures determined for the pAPN ectodomain alone (not shown), crystallized using distinct conditions. The pAPN molecular assembly shown here might thus be representative of the dimer described for mammalian APN on membrane surfaces [33].

### The RBD-pAPN binding interface

In the crystals of the PRCV RBD-pAPN complex, the RBD tip contacts a membrane-distal region of the pAPN ectodomain (Figure 3A). The conformations of the receptor-binding loops (β1–β2 and β3–β4) at the tips of the two PRCV β-barrel domains in the structure are identical (Figure S1B), suggesting very similar RBD-pAPN interactions in both complexes of the asymmetric unit. The virus-receptor interaction buried ∼870 Å^2^ of the virus protein, 60% of which corresponded to the β1–β2 region (Figure 3B) and 30% to the β3–β4 turn (Figure 3C). The size of the pAPN surface buried by the RBD was similar (∼770 Å^2^), and included pAPN residues ranging from alpha helix 19 (α19) to 22 (α22) in domain IV, and a few domain II residues (Figure S2, Table S2).

The end of the pAPN helix α19 and helix α21 contacted the β1–β2 region of the RBD (Figure 3B). The Tyr side chain (Tyr528 in TGEV), which protrudes at the β-turn in PRCV and TGEV RBDs (Figure 3B and 3D), is almost fully buried in the complex, locating between the first N-acetyl glucosamine (NAG7361) linked to pAPN Asn736, the end of helix α19, and the first half of helix α21 (Figure 3B). The hydroxyl group of the RBD Tyr528 was hydrogen bonded to side chains of pAPN residues Glu731 and Trp737, and contributed to virus-receptor binding specificity. The preceding RBD Gly527 residue was at the pAPN proximal side of the β-turn, hydrogen bonded to the pAPN Asn736 main chain; at the opposite side, the RBD Gln530 side chain formed a network of hydrogen bond interactions with pAPN NAG7361 and Asn736 side chain (Figure 3B). The N-acetyl moiety of the glycan also interacted with RBD residues at the β2 and β6 strands (Figure 3B, Table S2). The pAPN N-linked glycan and surrounding residues that contact the CoV RBD β1–β2 region in the structure were identified as one of the APN determinants of the CoV host range [20].

The second relevant virus-receptor interacting region engaged a β-turn at the beginning of the RBD β3–β4 loop (Figure 3C and 3D). The unique RBD Trp571 residue, which protrudes at the turn, docked in a pAPN cavity formed by the coils that precede helices α22 in domain IV and α5 in domain II (Figure 3C and S2). The bulky side chain of the RBD Trp571 residue packed against pAPN residues His786 and Pro787, and its imino group was hydrogen bonded to the main chain carbonyl of Asn783 (Figure 3C). The RBD Trp571 as well as the RBD Tyr528 at the β-barrel tip in TGEV and PRCV appear to be central residues in the virus-receptor interaction, as they contact with many pAPN residues and contribute also to binding specificity by mediating polar interactions with the pAPN (Table S2).

**Figure 4:**
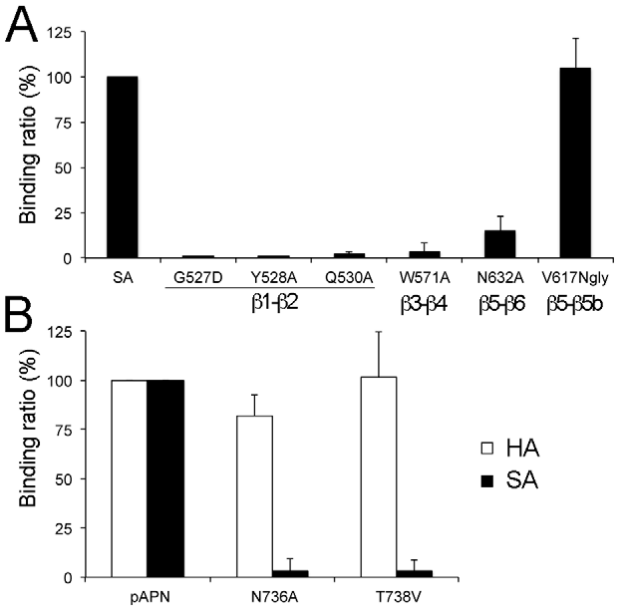
Important virus-receptor binding motifs. **A.** Binding of TGEV RBD mutants to cell surface pAPN. Relative binding (%) was determined for mutant to wild type SA proteins (see Materials and Methods). Substituted residues at the RBD tip that contact pAPN in the PRCV RBD-pAPN structure are shown in Figure 3, except for the V617Ngly mutant, with a glycan at RBD position 617 in the β5-β5b loop, outside the RBD tip (see Figure 3D). **B.** TGEV RBD binding to cell surface pAPN glycosylation mutants. Relative binding of the SA protein and the anti-HA mAb to HA-tagged pAPN proteins with (pAPN) or without the glycan linked to Asn736 (N736A and T738V). Mean and standard deviation for three experiments.

To confirm the contribution of the PRCV or TGEV RBD β-barrel tip in pAPN receptor recognition, we analyzed binding of wild type and mutant TGEV RBD proteins to cell surface-expressed pAPN (Figure 4A). Mutations in the three regions (β1–β2, β3–β4 and β5–β6) that build the receptor binding edge of the β-barrel decreased RBD binding to pAPN, whereas mutations outside the receptor-binding region (V617Ngly) had no effect on receptor recognition. Deletion of the pAPN Asn736 glycosylation site also abolished TGEV RBD binding to cell surface-expressed pAPN (Figure 4B). Deletion of the homologous glycan in feline APN similarly prevents cell infection by feline, canine and porcine CoVs, all of which share the glycan-binding Tyr residue in the β1–β2 turn (see below), whereas addition of this glycan to human APN is sufficient to render it a TGEV receptor [20].

### RBD structures in TGEV and in other CoVs

**Figure 5:**
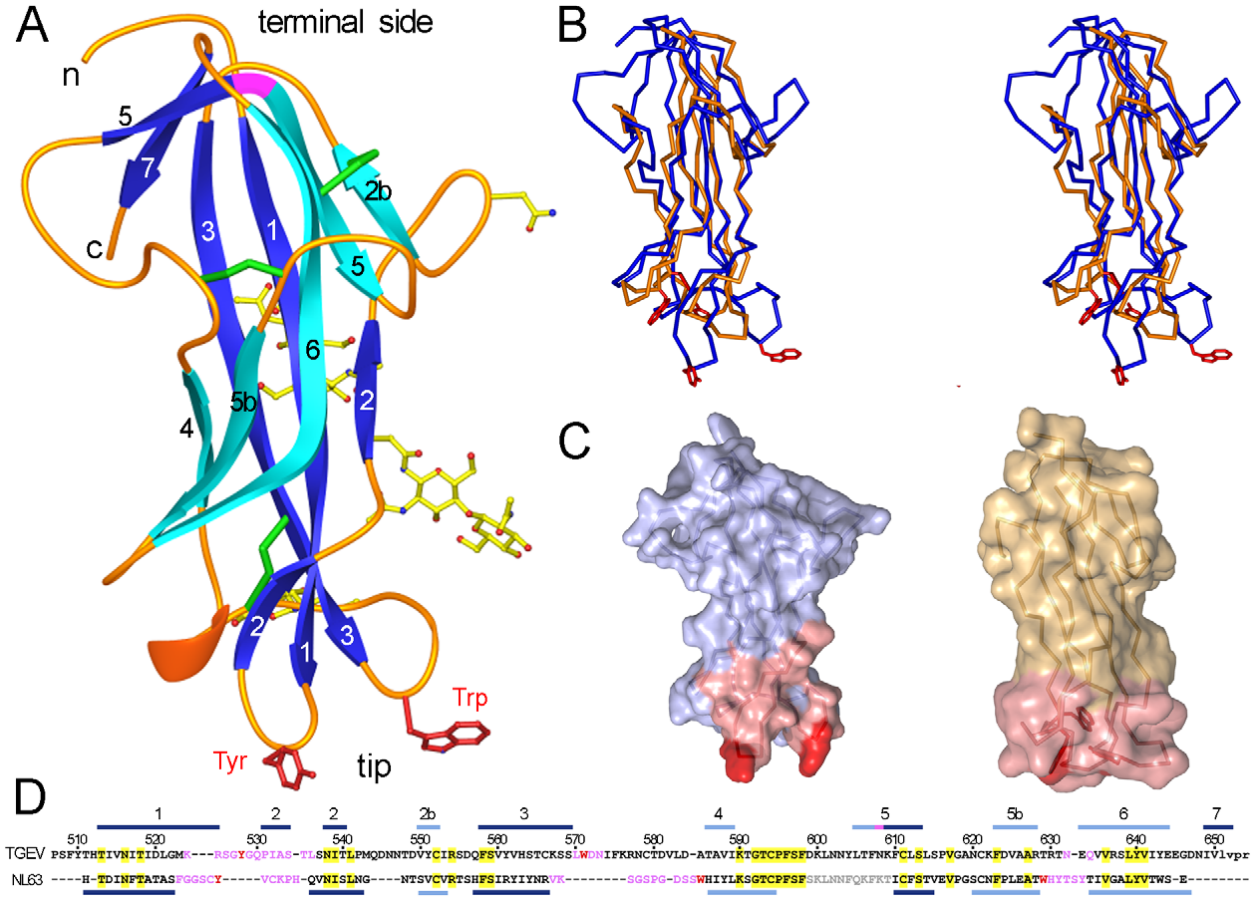
Structure of the RBD of TGEV and conformation of the receptor-binding edge in *Alphacoronavirus*. **A**. Ribbon diagram of RBD protein structure with β-strands in light or dark blue, coils in orange, and helix in red. A β-bulge at β-strand 5 is shown in magenta. N- and C-terminal ends on the terminal side of the structure are indicated in lowercase letters. The Asn residues at glycosylation sites and the attached glycans defined in the structure are shown as a ball-and-stick model, with carbons in yellow. Cysteine residues and disulfide bonds are shown as green cylinders. Side chains of the pAPN-binding Tyr and Trp residues in the loops at the β-barrel domain tip are shown in red in panels A to C. **B**. Stereo view of superimposed *Alphacoronavirus* RBD structures. The pAPN-binding RBD of TGEV is in blue and the ACE2-binding RBD of HCoV-NL63 (PDB ID 3KBH) is in orange. **C**. Surface representation of the TGEV and HCoV-NL63 RBD structures in B, with receptor-binding residues in pink or red. **D**. Structure-based sequence alignment of *Alphacoronavirus* RBD structures shown in C. β-strands are marked (bars) above or beneath their sequences. TGEV sequence is numbered. ACE2 receptor-binding residues reported for HCoV-NL63 [11], as well as pAPN receptor-binding residues for TGEV (Supplementary Table S2) are colored as in C. Residues absent in the RBD structures are in grey, and the thrombin recognition sequence at the end of the TGEV RBD is in lowercase letters.

We determined the crystal structures of the related TGEV and PRCV RBDs bound to two distinct ligands. The RBDs adopt β-barrel structures with small differences in the ligand binding loops (Figures S1). In the RBD, each of the two highly twisted β-sheets that build the β-barrel is formed by five β-strands (Figure 5A). The bent β-strand 5 (β5) crosses both β-sheets and has a β-bulge at Asn608 (Figure 5A, magenta). At one side of the β-barrel, all β-strands are antiparallel (Figure 5A, cyan), whereas on the opposite β-sheet, the β1 and β3 strands run parallel (Figure 5A, blue). N-linked glycans cluster at one side of the β-barrel (Figure 5A). N- and C-terminal ends of the RBD, where other S protein domains presumably lie, are opposite the ligand-binding tip of the β-barrel, where the pAPN-binding Tyr and Trp residues protrude (Figure 5A).

A DALI search of structural homologs showed the greatest similarity (Z score of 10) with the RBD of the ACE2 receptor-binding HCoV-NL63 (root-mean-square deviation of 2.4 Å for 103 residues), the other *Alphacoronavirus* RBD whose structure is known [11]. The cores of the TGEV and HCoV-NL63 β-barrel domains are structurally similar, but the loops at the tips (Figure 5B and 5D). The tip region of the HCoV-NL63 RBD is the ACE2 receptor-binding edge and has a “bowl”-shaped conformation (Figure 5C) that differs from the TGEV RBD protruding edge. Aromatic residues protrude from the β-turns at the tip of the β-barrel in TGEV, whereas they are partially buried at the center of the “bowl”-shaped edge in HCoV-NL63 (Figure 5B and 5C). The distinct RBD tip conformation in ACE2-binding HCoV-NL63 and in APN-binding TGEV might be a determinant of their distinct cell entry receptor specificities.

**Figure 6:**
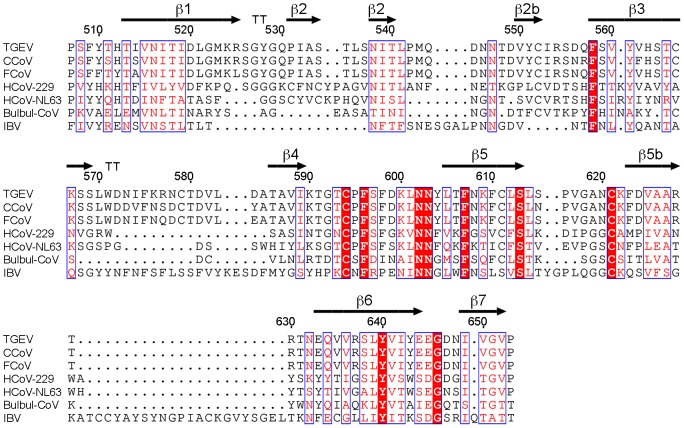
Sequence alignment of homologous CoV RBD. Alignment was carried out with the T-Coffee program (http://www.ebi.ac.uk/). RBD sequences of TGEV, canine and feline CoVs (*Alphacoronavirus* 1), human CoVs (*Alphacoronavirus*), Bulbul-CoV (putative *Deltacoronavirus*) and IBV (*Gammacoronavirus*). Sequence of the TGEV RBD is numbered and its β-strands are shown with arrows. Residues in the two turns at the tip of the TGEV RBD β-barrel structure are indicated with a double T (see Figure 5).

The degree of sequence identity in the RBD region among members in the species *Alphacoronavirus* 1 (∼90% identity) suggests a structure closely related to that of TGEV, including conformation of the receptor-binding loops (β1–β2 and β3–β4) at the β-barrel tip (Figure 6). Therefore, TGEV, PRCV, CCoV and FCoV must recognize the APN receptor in similar fashion. In contrast, the receptor-binding loops at the tip appear to have a different conformation from TGEV in the HCoV-229E RBD, which also binds to the APN. In this CoV, the β1–β2 region has two Cys, as in HCoV-NL63, and lacks the APN-binding Tyr residue in *Alphacoronavirus* 1, although it preserves the two Gly residues found in the TGEV β-turn (Figure 6). The β3–β4 loop in HCoV-229E is markedly shorter than in TGEV, but it also has a Trp residue. Sequence identities between the RBD of TGEV and IBV (*Gammacoronavirus*) or the Bulbul-CoV (tentative *Deltacoronavirus*) are relatively large (∼25%), and similarities are found mostly in β-strands and at the RBD C-terminal half (Figure 6). These data indicate a conserved RBD fold between *Alphacoronavirus* and *Gamma-* or *Deltacoronavirus*. There is less sequence similarity between the *Alpha-* and *Betacoronavirus* RBD regions (∼10%), which correlates with notable structural differences between their RBDs [8], [11], [13]. The RBDs of the SARS and MHV *Betacoronavirus* adopt folds unrelated to the β-barrel shown for *Alphacoronavirus*.

### The pAPN-binding edge of the TGEV RBD is the main determinant of antigenic site A

**Figure 7:**
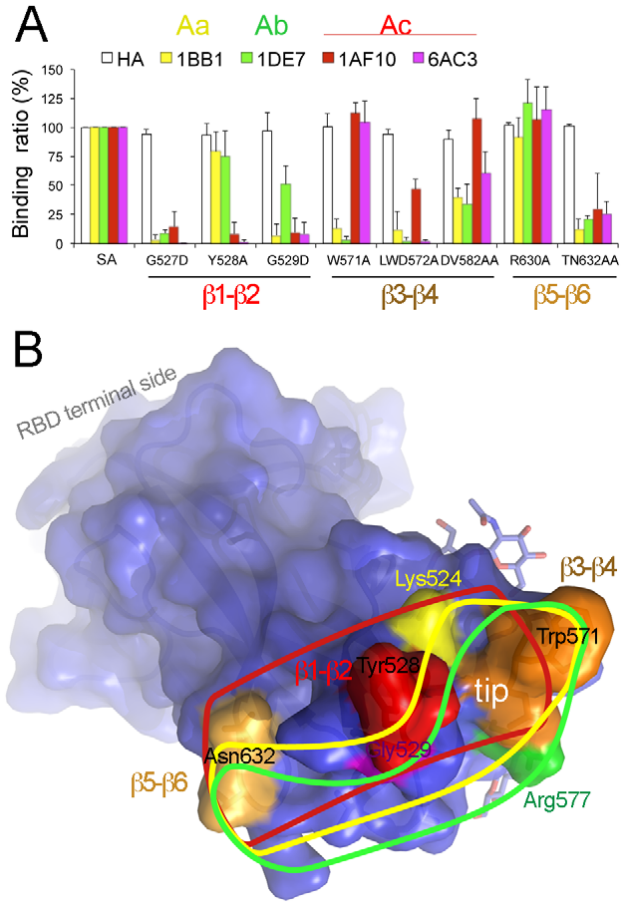
Determinants of TGEV S antigenic site A. **A.** Binding of TGEV-neutralizing, site A-specific mAbs to RBD mutants. Relative binding (%) of mutants to wild type SA protein is shown for TGEV S-specific mAbs (top; described in Figure 1C) and a control anti-HA antibody (see Materials and Methods). RBD regions in which mutations locate are shown (bottom; see also Figure 5D). Mean and standard deviation of data from at least three experiments. **B.** Antigenic site A in the TGEV RBD and epitopes for antibodies. Surface and ribbon representation of the RBD with the 1AF10 contact regions colored as in Figure 2B. Three antibody-binding residues (Tyr528, Trp571 and Asn632) in the loops at the RBD tip, as well as TGEV Lys524, Arg577 and Gly529 residues associated with Aa, Ab and Ac subsites [24], respectively. Lines indicate epitopes for mAbs specific for each of the three antigenic subsites: Aa in yellow, Ab in green and Ac in red.

The most TGEV-neutralizing mAbs, including 1AF10, recognize antigenic site A in the S protein, divided into the Aa, Ab and Ac subsites [24]. To further characterize site A antigenic determinants in the TGEV RBD, we mutated RBD residues targeted by the 1AF10 mAb (Figure 2) and some surrounding residues, and analyzed binding to other site A-specific mAbs. The antigenicity of residues in the β1–β2 region, in the center of the epitope for 1AF10 (Figure 2C), was determined by monitoring mAb binding to RBD mutants with TGEV residue substitutions Gly527 (G527D), Tyr528 (Y528A) and Gly529 (G529D) (Figure 7A). All three substitutions abolished RBD binding by the Ac subsite-specific mAbs 1AF10 and 6AC3. The Y528A RBD mutant was recognized by Aa- (1BB1) and Ab-specific (1DE7) mAbs (Figure 7A), and mAb 1DE7 also bound the G529D mutant.

In contrast to the antibody binding profile of the Y528A RBD mutant, Ala substitution of the TGEV Trp571 residue (W571A), a pAPN-binding residue in the β3–β4 loop at the periphery of the RBD epitope for 1AF10 (Figure 2C), did not affect binding by the Ac-specific mAbs (1AF10 and 6AC3), whereas RBD recognition by 1BB1 and 1DE7 mAbs was greatly reduced (Figure 7A). Deletion of the β3–β4 turn (LWD572A mutant) reduced 6AC3 mAb binding to the RBD markedly, with a partial reduction in 1AF10 binding (Figure 7A); this indicates that mAb 6AC3 recognizes a broader epitope, which correlates with its higher TGEV neutralization activity [25]. Replacement with Ala of RBD residues Thr631 and Asn632 at the β5–β6 hairpin, which contacts the 1AF10 mAb in the RBD-1AF10 structure (Figure 2C), reduced binding by all site A-specific mAb (Figure 7A). This might be a result of a conformational effect induced on the nearby β1–β2 region of the RBD.

Results for antibody binding to RBD mutants showed that site A epitopes extend across the TGEV RBD tip, although there are some differences among the three A subsites (Figure 7B). The epitopes recognized by Aa- and Ab-specific mAbs bear the exposed TGEV Trp571 residue at the β3–β4 loop, whereas epitopes for the Ac-specific mAbs center on Tyr528 in the β1–β2 turn. None of the mAb tested simultaneously targeted the two aromatic side chains (Tyr and Trp) at the tip of the TGEV RBD that bind to the pAPN. Subsite-specific residues defined by mar mutants (Lys524 for Aa, Arg577 for Ab and Gly529 for Ac) might be located at the periphery of their respective epitopes (Figure 7B). Ab and Ac subsites appear to be relatively far apart, with the Aa epitope in an intermediate position. The RBD tip, shown here as the pAPN-binding edge of the domain (Figure 3), is the main S protein determinant of antigenic site A, recognized by the most effective neutralizing antibodies of TGEV and related CoV infections [25], [26].

## Discussion

Here we show how a group of CoVs attaches to the cell surface APN metalloprotease for entry into host cells, and how some CoV-neutralizing antibodies prevent infection. The RBD-receptor complex structures determined for *Alphacoronavirus* indicate that the conformation of the receptor binding edge in the envelope S proteins probably determines their receptor-binding specificity. The CoV that bind APN analyzed here have protruding receptor-binding motifs that engage recessed surfaces on the receptor. This mode of receptor recognition is essentially opposite to that reported for CoV binding to the ACE2 receptor, where recessed receptor-binding motifs in the viral RBD cradle exposed surfaces of the ACE2 ectodomain [11], [13]. In the case of pAPN, an N-linked glycan is also engaged in the virus-receptor interaction. The inherent flexibility of this glycan might facilitate the initial contact of the CoV Tyr residue with APN amino acids, and subsequent virus-receptor interactions could lock the bound Tyr between the glycan and an α-helix (Figure 3B). The glycan N-linked to Asn736 in pAPN is also conserved in canine and feline APN proteins (Figure S2), as are the viral S protein residues that interact with this glycan in the RBD β1–β2 and the β5–β6 regions (Figure 6). This unique glycan-virus interaction must thus be conserved among the different CoVs in the species *Alphacoronavirus* 1, in accordance with the glycan requirement reported for cell infection by CCoV, FCoV, and TGEV/PRCV [20]. The lack of this glycan in human APN (Figure S2) and the absence of the interacting Tyr residue in the β1–β2 region of HCoV-229E RBD (Figure 6) imply distinct virus-APN local contacts in humans. As shown for the *Alphacoronavirus* 1 group, however, HCoV-229E probably has a protruding receptor-binding edge in the envelope S, responsible for its APN-binding specificity.

The structure of the RBD-1AF10 complex, together with structure-guided RBD mutagenesis and mAb binding data, demonstrated that the receptor-binding region is a major antigenic determinant in the envelope S protein of CoV that bind APN. Potent TGEV-neutralizing antibodies, such as the 6AC3 mAb [25], target key APN-binding residues in the S (Figure 7), preventing infection. Data from antibody neutralization-resistant TGEV mar mutants nonetheless show that some substitutions can be accommodated in the receptor-binding region of *Alphacoronavirus*, which confer the ability to escape immune neutralization, while preserving the receptor-binding affinity necessary for cell entry [24], [26]. Our results thus demonstrate that the receptor-binding region in *Alphacoronavirus* is under selective pressure from the immune system, as described for other viruses [34], [35], [36], [37]. It is tempting to speculate that immune pressure on exposed receptor-binding residues in the CoV S could lead to conformational changes in receptor-binding edges of CoV RBDs. This would result either in changes in the APN-recognition mode observed with HCoV-229E and TGEV, or in conformational changes in the RBD tip that lead to a receptor specificity switch for cell entry, as observed for HCoV-NL63 [11]. Virus use of recessed binding regions, as for HCoV-NL63, is a well-defined strategy for hiding conserved receptor-binding residues from antibodies [34], [36]. Like HCoV-NL63, SARS-CoV uses a recessed, although broader ACE2-binding surface, which can accommodate mutations that permit cross-species receptor recognition [13]. It remains to be understood why, despite major changes in the receptor-binding region, all these CoV use metalloproteases as cell entry receptors.

In the course of our studies, we also determined the crystal structure of the cell surface APN, an important target for cancer therapies. The domain architecture of APN resembles that of related aminopeptidases [30], [31], [32]. Here we show a unique dimer configuration for the APN, mediated by its domain IV, the most divergent domain among M1 aminopeptidases [31]. The implication of these structural findings for APN biology will require further biochemical analysis. Knowledge of the structure is leading to research on the mechanism of action of numerous anti-tumor compounds that target mammalian APN [19]; these studies will be fundamental for improving drug specificity. The detailed view of the APN-CoV interaction shown here might also lead to development of small molecules to block CoV infection. We have identified the receptor-binding region as the major antigenic site in the *Alphacoronavirus* envelope S, which could guide the design of immunogens that boost CoV-neutralizing immune responses to key motifs for virus cell entry.

## Materials and Methods

### Recombinant protein preparation

Design of soluble S proteins variants of TGEV and PRCV has been described [27]. The SA protein containing the RBD of TGEV was derived from the SC11 strain, and contains residues 481 to 650 of the TGEV S, an N-terminal influenza hemagglutinin HA peptide, and either a FLAG mAb epitope (monovalent SA-Flag variant) or the human IgG1 Fc portion (bivalent SA-Fc variant) at the C-terminal end. The engineered soluble pAPN contains residues 36 to 963 (ectodomain) of the cell surface protein fused to HA and FLAG tags at the N and C terminus, respectively [27]. The soluble S protein crystallized in complex with the pAPN was derived from the PRCV HOL87 strain (S3H in [27]), and contains the N-terminal 426 residues of the PRCV S protein and same C-terminus as the TGEV-derived SA protein [27]. A recombinant membrane bound pAPN with an HA tag at the C-terminal end was engineered for cell surface expression. Thrombin recognition sequences were introduced between the tags and the viral or pAPN protein sequences.

Proteins were produced in transiently transfected 293T or stably transfected CHO-Lec 3.2.8.1 (CHO-Lec) cells as described [27], and concentration in cell supernatants determined by ELISA. Proteins prepared in CHO-Lec cells were used in crystallization experiments. Hybridoma cells secreting the TGEV S mAbs were grown in DMEM supplemented with 10% FCS in roller bottles. Proteins secreted to culture supernatants were initially purified by affinity chromatography. All protein samples were further purified by size exclusion chromatography in HEPES-saline buffer (20 mM HEPES, 150 mM NaCl) pH 7.5.

The Fab fragment of the 1AF10 mAb was prepared by papain digestion of the purified antibody. The reaction was terminated by the addition of E64 (Sigma) and the Fab fragment purified by size exclusion and ion exchange chromatography using HEPES-saline buffer pH 8.0. The polypeptide chains of the Ig variable domains of the 1AF10 mAb were determined by sequencing of their cDNA prepared from reverse transcribed mRNA purified from hybridoma cells.

### Antibody and pAPN binding assays

Binding of anti-TGEV S or -HA (control) mAb to wild type and mutant SA proteins was tested in 96-well plates, using purified mAb or hybridoma supernatants. The SA-Fc fusion proteins in serum-free (opti-MEM, Invitrogen) cell supernatants were bound to plastic, and mAb binding monitored by optical density (OD_490 nm_). At least four SA-Fc protein concentrations ranging from 10 to 1 µg/ml were used in duplicate and average binding determined in each experiment. Binding ratios were determined after correction for background binding.

APN binding assays were also carried out with the SA-Fc fusion protein comprising the TGEV RBD. BHK-pAPN cells constitutively expressing cell surface pAPN were used for binding experiments comparing wild type and mutant RBDs, whereas transiently transfected 293T cells were used for analysis of RBD binding to pAPN glycosylation mutants. Binding was monitored as the percentage of stained cells with the Fc fusion proteins and FITC labeled anti-Fc antibodies by Fluorescence-Activated Cell Sorting (FACS), as shown in Figure 1B. The percentage of cells stained was determined for each protein sample and corrected for background staining. pAPN binding ratios for wild type and mutant RBD proteins shown in Figure 4A were determined from the percentage of BHK-pAPN cells stained with same concentration of wild type and mutant SA-Fc proteins. The binding ratios for wild type and mutant pAPN glycosylation mutants shown in Figure 4B were determined from the percentage of SA-Fc stained 293T cells expressing similar amounts of HA-tagged pAPN proteins. Cell surface expression of the pAPN-HA protein was determined with the HA 12AC5 mAb.

### Protein and complex crystallization

The TGEV RBD in complex with the 1AF10 Fab fragment was crystallized using the size exclusion-purified complex of a monovalent SA-Flag protein containing the TGEV RBD and the mAb fragment. Crystals of the complex were prepared by the hanging drop method with a 20 mg/ml protein sample and a crystallization solution of 16% PEG-4K, 0.2 M NaAc, 0.1 M 1,2,3-octanetriol isomer T and 0.1 M Tris buffer pH 8.5. Crystals were frozen with crystallization solution containing 20% ethylene glycol. Diffraction data extending to 3 Å resolution were collected at the ID29 beamline (TGEV RBD-1AF10 in Table 1).

Crystallization of the pAPN ectodomain in complex with porcine CoV S proteins was carried out with mixtures of the receptor protein and several TGEV and PRCV protein variants comprising the receptor-binding region (SA, S1H and S3H in [27]). Crystals appeared only in trials performed with an equimolar mixture of pAPN and the S3H protein derived from the PRCV S at a final protein concentration of 13 mg/ml, and with a crystallization solution of 20% PEG-4K, 0.2 M lithium sulfate and 0.1 M Tris buffer pH 8.5. Crystals were transferred to crystallization solution containing 20% ethylene glycol and frozen for diffraction data collection at the ID29 beamline (PRCV RBD-pAPN in Table 1).

### Structure determination

The structure of the TGEV RBD-1AF10 Fab fragment was initially determined by the molecular replacement (MR) method using the PHASER program [38], and two search models having either the variable or constant regions of the PDB ID 1AIF mAb structure. The 1AF10 Fab model structure was built manually following electron density maps determined from the MR solution, after improvement with the DM program [39]. The 1AF10 Fab structure was refined with the program phenix.refine [40], which provided an excellent electron density map for building residues 507 to 650 of the TGEV S, as well as four residues of a thrombin recognition site at the C-terminus. Final structure refinement of the complex was carried out with data extending to 3.0 Å resolution (statistics in Table 1). Three cycles of solvent correction, refinement of individual coordinates and atomic displacement parameters combined with TLS were applied in each step of structure refinement with phenix.refine, which was alternated with manual adjustment of the model to the electron density maps. All residues are in allowed regions of the Ramachandran plot. SA protein residues included in the structure of the TGEV RBD are shown in Figure 3D.

The structure of the PRCV RBD-pAPN complex was resolved by the MR method using the pAPN structure determined alone (manuscript in preparation) and the TGEV RBD structure as search models. MR solutions were obtained for the two pAPN molecules (chains A and B) of the asymmetric unit and for one RBD molecule (chain E). The three molecules were adjusted manually and refined with the phenix.refine program. The second RBD molecule (chain F) bound to pAPN molecule B was built manually into the electron density map. The 282 residues N-terminal to the PRCV RBD in the S3H protein were largely disordered or degraded during crystallization, and are absent in the structure. The complex structure was refined with the program phenix.refine applying solvent correction, NCS, refinement of individual coordinates and atomic displacement parameters combined with TLS (Table 1). The current model comprises residues 60 to 963 of the pAPN ectodomain with a zinc metal ion at the pAPN enzyme active site, and residues 283 to 426 of the PRCV S, homologous to the TGEV S residues 507 to 650 that defined the TGEV RBD structure (Figure 3D). All the residues are in allowed regions of the Ramachandran plot.

Coordinates and structure factors have been deposited in the Protein Data Bank with ID codes 4F2M (TGEV RBD-1AF10) and 4F5C (PRCV RBD-pAPN).

### Analysis and representation of crystal structures

Buried surfaces and residues at the molecular complex interfaces were determined with the PISA server (http://www.ebi.ac.uk/msd-srv/prot_int/pistart.html). Only residues with at least 10% of their surface buried at interfaces in the two independent molecules of the crystal asymmetric units are shown. Figure 2D was prepared with LIGPLOT (http://www.ebi.ac.uk/thornton-srv/software/LIGPLOT/), Figure 5A with Ribbons [41] and the other structure representations with PyMOL (pymol.org). Structural alignments were carried out with Modeller using a gap penalty of 3 [42].

### Protein sequences

Accession numbers of the *Alphacoronavirus* S proteins mentioned are Q0PKZ5 (TGEV), Q65984 (CCoV), P10033 (FCoV), P15423 (HCoV-229), Q6Q1S2 (HCoV-NL63), B6VDW0 (Bulbul-CoV) and Q9Q9P1 (IBV). The PRCV HOL87 S protein sequence is reported in reference [21]. Sequence identities among S proteins were determined with psiblast (http://www.ebi.ac.uk/Tools/sss/psiblast/). Accession number for the pAPN protein is P15145.

## Supporting Information

Figure S1 **Structures of TGEV and PRCV RBDs.** **A.** Secondary structure elements of the RBD structures. β-strands are shown with arrows and colored in blue and cyan, a β-bulge at the β-strand 5 is shown in magenta, helix with a red cylinder, coils with black lines, and disulphide bonds with green lines. **B.** Stereo view of the superimposed asymmetric unit RBD structures of TGEV (blue and cyan), complex with the 1AF10 mAb, and of PRCV (green and red), complex with the pAPN protein. View as in Figures 2A and 5A. Locations of N and C terminal ends are indicated in lowercase letters.
(TIF)

Figure S2 **Mammalian APN ectodomains.** Sequence alignment of the porcine, canine, feline and human APN proteins with conserved residues highlighted in red. Secondary structure elements of the pAPN structure determined in complex with the RBD of PRCV are shown above the sequences. CoV-binding residues and those engaged in pAPN dimerization are highlighted in blue and green, respectively, whereas those at the pAPN catalytic site are in yellow. Residues coordinating the zinc ion are marked with an asterisk, and the N-linked glycosylation site recognized by CoV is marked with a triangle at the pAPN Asn736. The beginning of each of the four APN domains is indicated.
(TIF)

Figure S3 **Aminopeptidases active site.** Side chains of residues at the catalytic site of four structurally aligned zinc aminopeptidases based on domain II are shown with stick representation, and with the coordinated zinc ion as a cyan sphere. Human ERAP-1 (PDB code 2XDT) is shown in green, aminopeptidase N of E. Coli (PDB code 2HPT) in magenta, aminopeptidase N of Neisseria meningitidis (PDB code 2GTQ) in blue, and pAPN in yellow. The glutamic acid located in the GAMEN motif is labeled in blue and those located at the conserved HExxHx_18_E motif are in red (sequence in Figure S2).
(TIF)

Figure S4 **Dimerization of the pAPN ectodomain in solution.** Size exclusion chromatography of the soluble pAPN ectodomain. Continuous line shows optical density (OD) at 280 nm for the elution volume. pAPN protein was run through a Superdex 200 16/60 column (GE Healthcare) with HEPES-saline buffer pH 7.5. Exclusion volume and size (kDa) of molecular weight markers are indicated. Determined molecular weight for the single recombinant glycosylated pAPN ectodomain is about 130 kDa, whereas the protein elutes with a volume corresponding to ∼300 kDa.
(TIF)

Table S1 **Sequence of homologous CDR-H3 loops in known mAb structures.** Sequence of homologous heavy chain CDR-H3 loops to that of the 1AF10 mAb, identified by a Blast search among protein structures, whose PDB codes are shown.
(TIF)

Table S2 **Intermolecular contacts in the PRCV RBD-pAPN complex structure.** RBD and pAPN residues in close contact (≤5 Å) in the two complexes of the crystal asymmetric unit, computed with the program NCONT [39]. RBD residues from the β1–β2, β3–β4 and β5–β6 regions at the tip of the β-barrel domain are shown, with those engaged in hydrogen bonding in red. TGEV/PRCV numbering is given for the RBD residues.
(TIF)
