# Supplementary figures and images for: Structural Bases of Coronavirus Attachment to Host Aminopeptidase N and Its Inhibition by Neutralizing Antibodies
Source: PLoS Pathog. 2012 Aug 2;8(8):e1002859. doi: 10.1371/journal.ppat.1002859 (PMC3410853; doi:10.1371/journal.ppat.1002859)

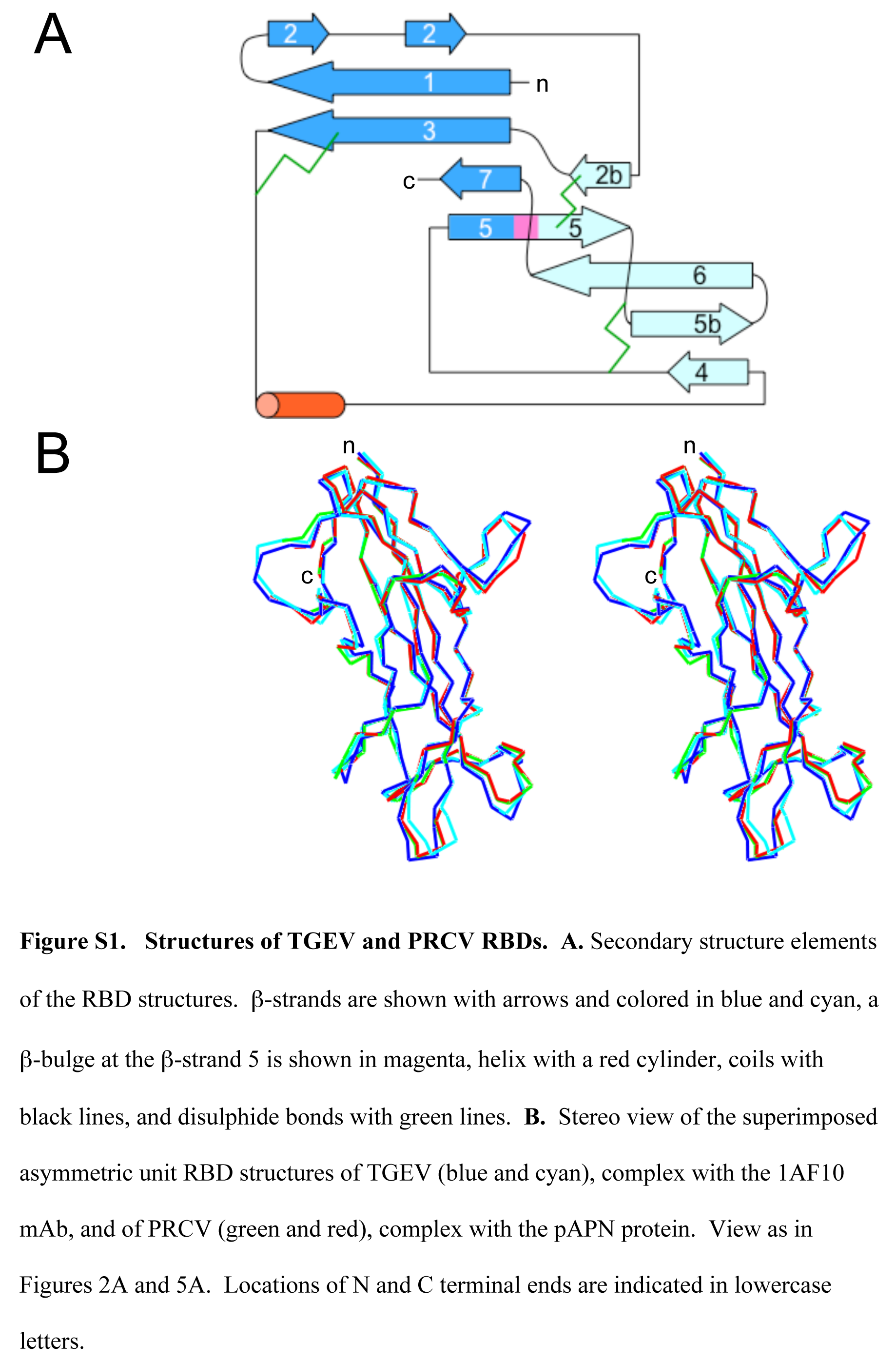

Supplement: Figure S1 — Structures of TGEV and PRCV RBDs. A. Secondary structure elements of the RBD structures. β-strands are shown with arrows and colored in blue and cyan, a β-bulge at the β-strand 5 is shown in magenta, helix with a red cylinder, coils with black lines, and disulphide bonds with green lines. B. Stereo view of the superimposed asymmetric unit RBD structures of TGEV (blue and cyan), complex with the 1AF10 mAb, and of PRCV (green and red), complex with the pAPN protein. View as in Figures 2A and 5A. Locations of N and C terminal ends are indicated in lowercase letters. (TIF) [file ppat.1002859.s001.tif]

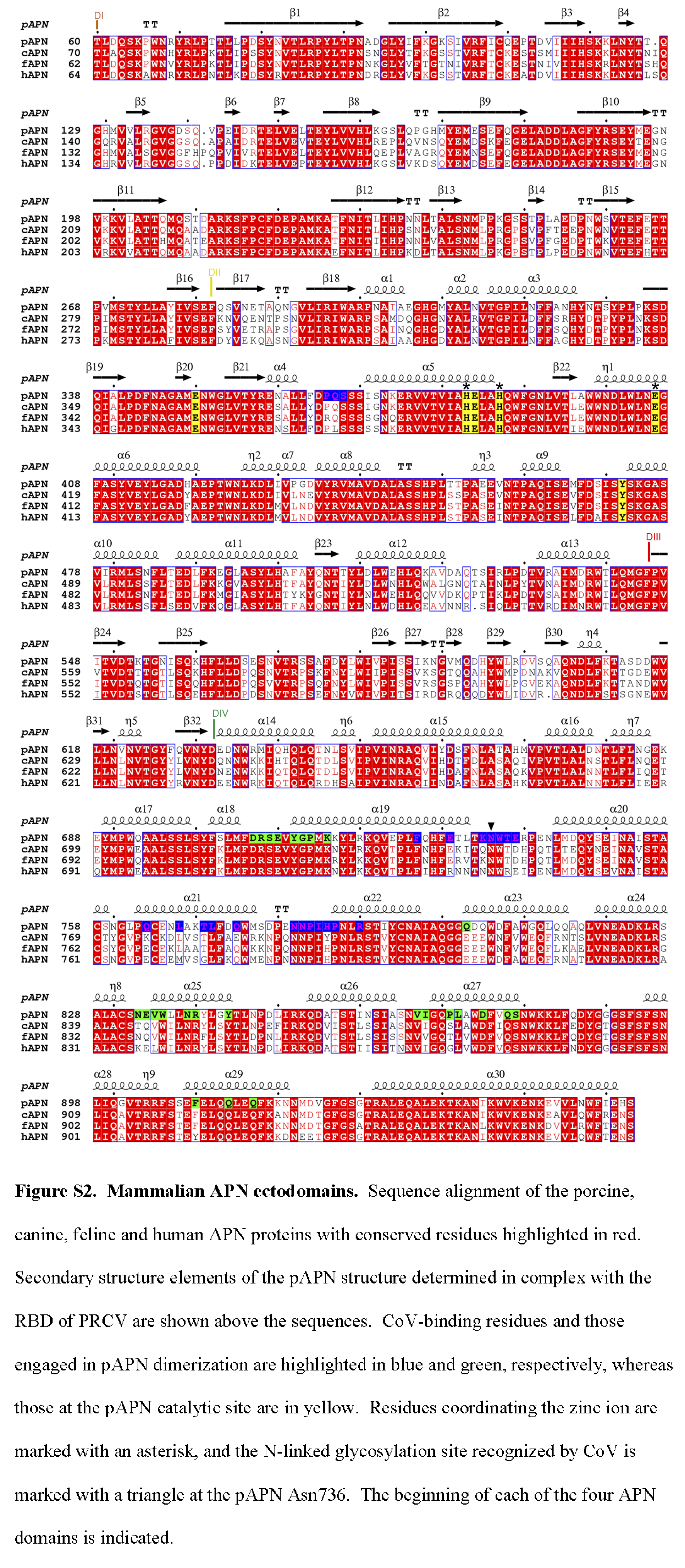

Supplement: Figure S2 — Mammalian APN ectodomains. Sequence alignment of the porcine, canine, feline and human APN proteins with conserved residues highlighted in red. Secondary structure elements of the pAPN structure determined in complex with the RBD of PRCV are shown above the sequences. CoV-binding residues and those engaged in pAPN dimerization are highlighted in blue and green, respectively, whereas those at the pAPN catalytic site are in yellow. Residues coordinating the zinc ion are marked with an asterisk, and the N-linked glycosylation site recognized by CoV is marked with a triangle at the pAPN Asn736. The beginning of each of the four APN domains is indicated. (TIF) [file ppat.1002859.s002.tif]

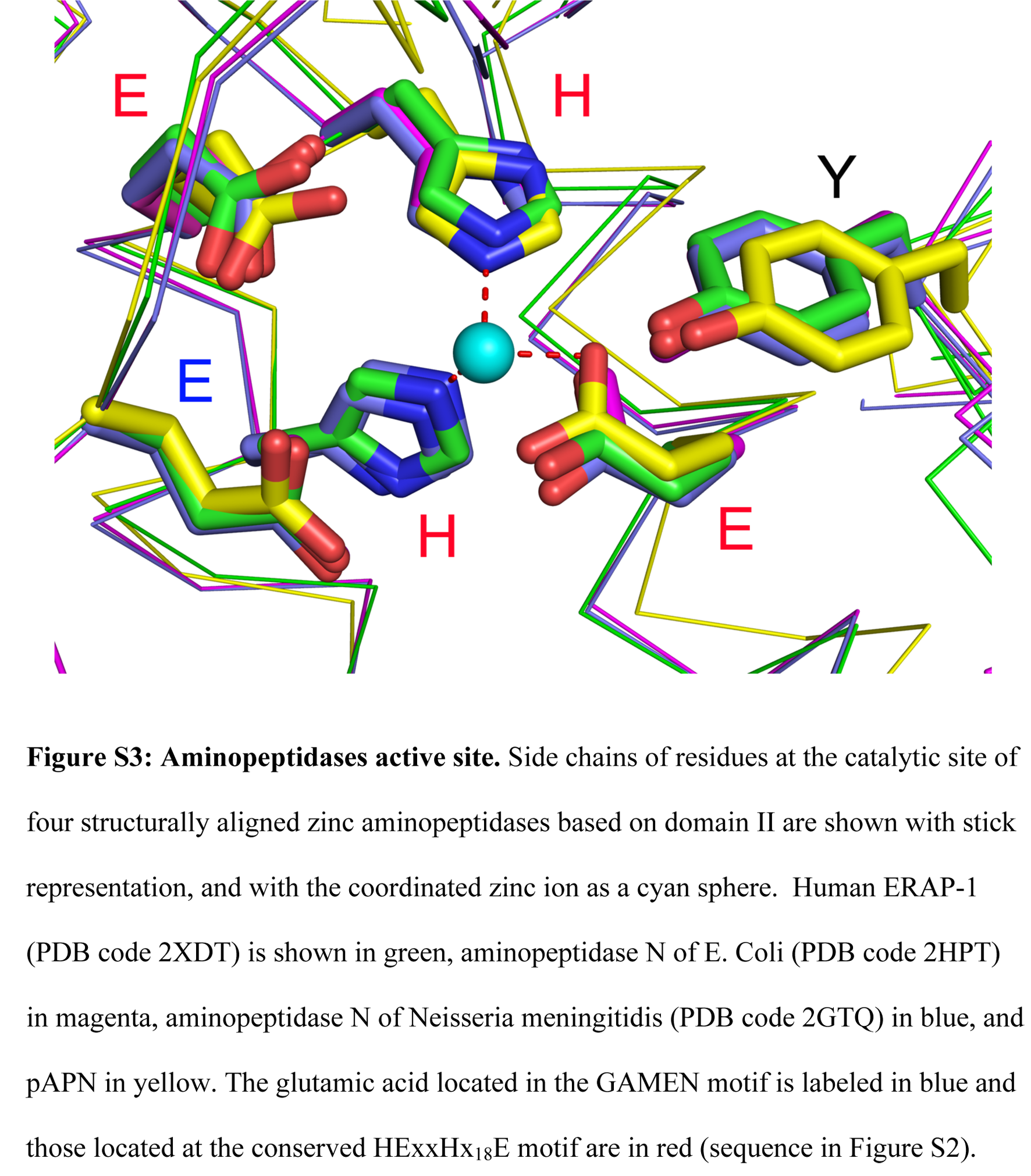

Supplement: Figure S3 — Aminopeptidases active site. Side chains of residues at the catalytic site of four structurally aligned zinc aminopeptidases based on domain II are shown with stick representation, and with the coordinated zinc ion as a cyan sphere. Human ERAP-1 (PDB code 2XDT) is shown in green, aminopeptidase N of E. Coli (PDB code 2HPT) in magenta, aminopeptidase N of Neisseria meningitidis (PDB code 2GTQ) in blue, and pAPN in yellow. The glutamic acid located in the GAMEN motif is labeled in blue and those located at the conserved HExxHx18E motif are in red (sequence in Figure S2). (TIF) [file ppat.1002859.s003.tif]

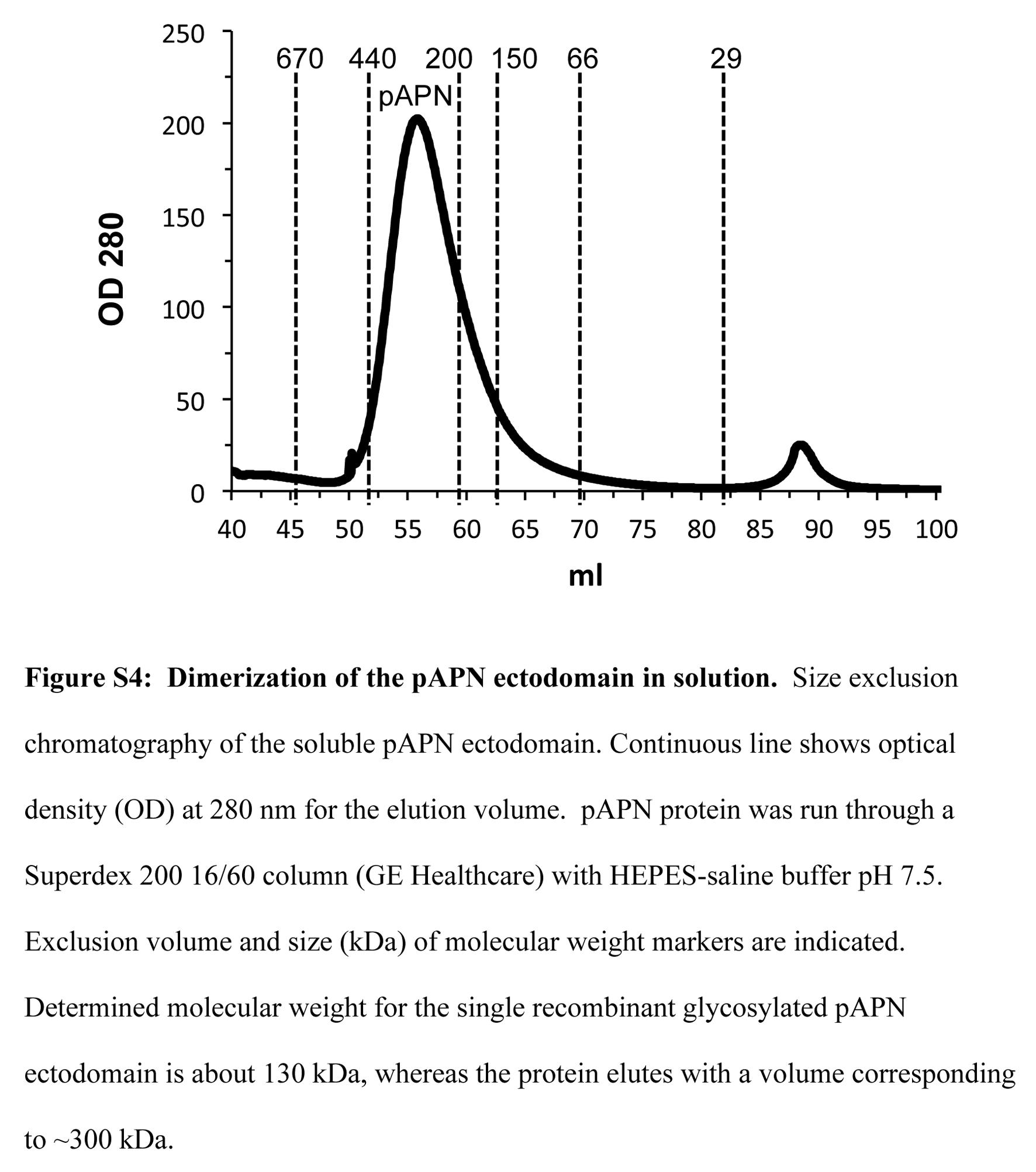

Supplement: Figure S4 — Dimerization of the pAPN ectodomain in solution. Size exclusion chromatography of the soluble pAPN ectodomain. Continuous line shows optical density (OD) at 280 nm for the elution volume. pAPN protein was run through a Superdex 200 16/60 column (GE Healthcare) with HEPES-saline buffer pH 7.5. Exclusion volume and size (kDa) of molecular weight markers are indicated. Determined molecular weight for the single recombinant glycosylated pAPN ectodomain is about 130 kDa, whereas the protein elutes with a volume corresponding to ∼300 kDa. (TIF) [file ppat.1002859.s004.tif]

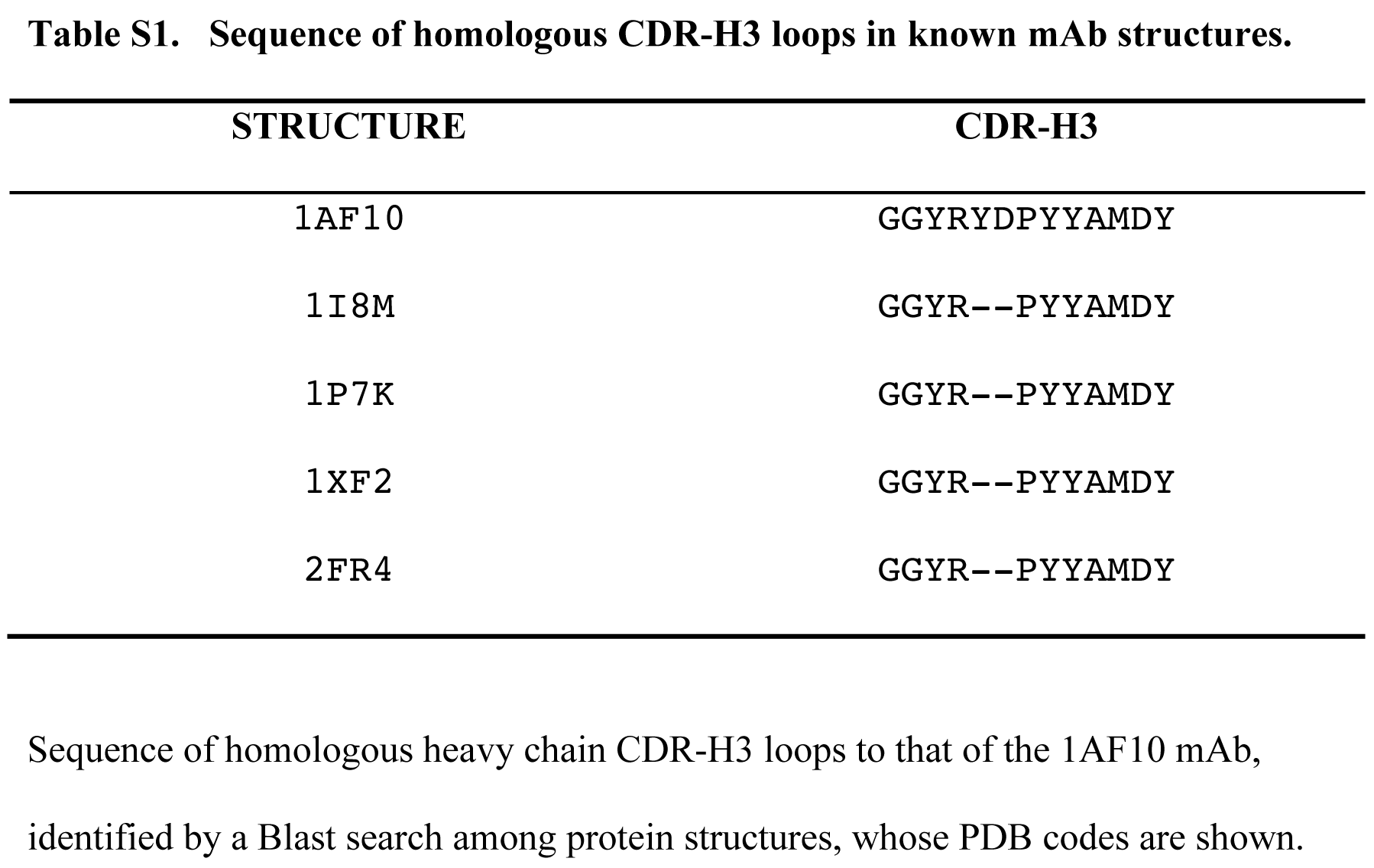

Supplement: Table S1 — Sequence of homologous CDR-H3 loops in known mAb structures. Sequence of homologous heavy chain CDR-H3 loops to that of the 1AF10 mAb, identified by a Blast search among protein structures, whose PDB codes are shown. (TIF) [file ppat.1002859.s005.tif]

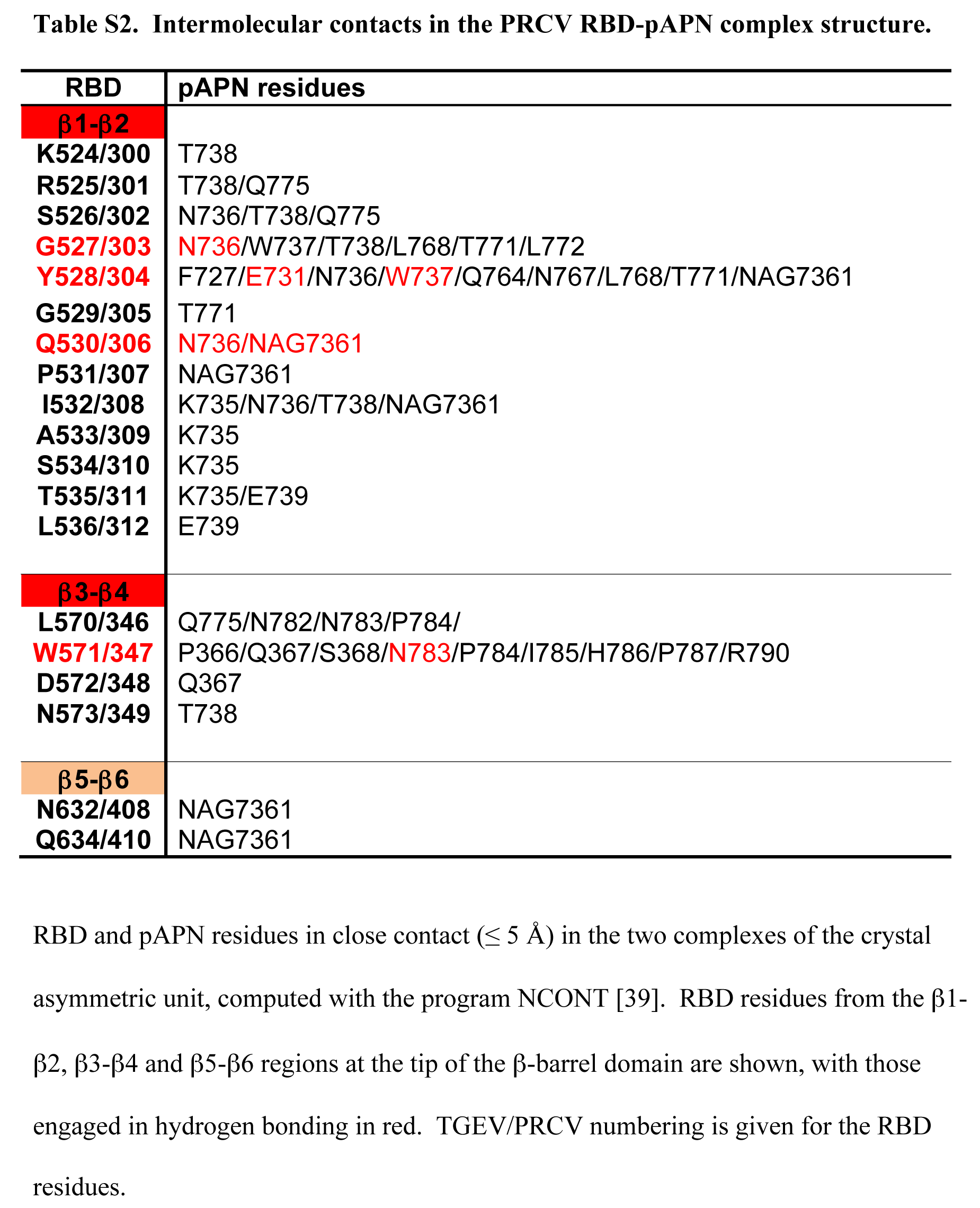

Supplement: Table S2 — Intermolecular contacts in the PRCV RBD-pAPN complex structure. RBD and pAPN residues in close contact (≤5 Å) in the two complexes of the crystal asymmetric unit, computed with the program NCONT [39]. RBD residues from the β1–β2, β3–β4 and β5–β6 regions at the tip of the β-barrel domain are shown, with those engaged in hydrogen bonding in red. TGEV/PRCV numbering is given for the RBD residues. (TIF) [file ppat.1002859.s006.tif]
